# Comparing the characteristcs of hospitalized patients admitted in involuntary or voluntary treatment after first episode psychosis

**DOI:** 10.1192/j.eurpsy.2024.649

**Published:** 2024-08-27

**Authors:** F. Leitão, S. A. Pinho, S. Sousa, J. Loureiro, C. Cunha, J. R. Silva, G. França, N. Oliveira, P. M. Ferreira, A. M. Moreira

**Affiliations:** ^1^ Serviço de Saúde Mental e Comunitária do Porto Ocidental; ^2^Serviço B, Hospital de Magalhães Lemos, Centro Hospitalar e Universitário de Santo António, Porto, Portugal

## Abstract

**Introduction:**

Individuals experiencing psychotic symptoms often lack insight into their conditions, especially in first psychotic episodes. According to the Portuguese Mental Health Law, involuntary hospitalization may be necessary in cases of severe mental disorder, involving a threat to the patient or his/her legal assets, when there is a refusal of the necessary treatment.

**Objectives:**

The aim of our study was to characterize patients admitted involuntarily for first psychotic episode and to compare them with the patients undergoing inpatient voluntary treatment.

**Methods:**

Out of a total of 87 patients diagnosed with first psychotic episode, hospitalized between 2020 and 2022 in our service, at *Hospital Magalhães Lemos*, 65 were included in the study. Exclusion criteria included patients from other residential areas. 40 patients were admitted under involuntary treatment, whereas 25 were hospitalized voluntarily. For both groups, we calculated the duration of untreated psychosis, the prevalence of psychoactive substance abuse, the type of treatment provided and the number of re-hospitalizations.

**Results:**

Patients in involuntary treatment had longer duration of untreated psychosis (71 vs 38 weeks). Among these patients, 53% had comorbid psychoactive substance abuse, in contrast with only 36% of voluntarily treated patients. Upon discharge, 58% of patients in involuntary treatment were prescribed depot antipsychotic medication, whereas only 12% of the ones in voluntary treatment. Out of 40 patients admitted involuntarily, 11 were re-hospitalized, but only 4 of the 25 patients in voluntary treatment (28 vs 16%).

**Conclusions:**

Patients in involuntary treatment probably suffered from more severe disease, as seen for the higher duration of untreated psychosis and frequent comorbid substance abuse. Injectable medication was the preferred choice at the time of discharge for this group. Additionally, they experienced higher rates of re-hospitalizations. Recent changes in Portuguese Mental Health Law, that aims to safeguard the rights and responsibilities of individuals with mental health care needs, motivated this study.

**Disclosure of Interest:**

None Declared

